# Dynamic Reconfiguration of the Supplementary Motor Area Network during Imagined Music Performance

**DOI:** 10.3389/fnhum.2017.00606

**Published:** 2017-12-12

**Authors:** Shoji Tanaka, Eiji Kirino

**Affiliations:** ^1^Department of Information and Communication Sciences, Sophia University, Tokyo, Japan; ^2^Department of Psychiatry, School of Medicine, Juntendo University, Tokyo, Japan; ^3^Department of Psychiatry, Juntendo Shizuoka Hospital, Shizuoka, Japan

**Keywords:** emotion, functional connectivity, imagery, imagination, mental simulation, language, representation

## Abstract

The supplementary motor area (SMA) has been shown to be the center for motor planning and is active during music listening and performance. However, limited data exist on the role of the SMA in music. Music performance requires complex information processing in auditory, visual, spatial, emotional, and motor domains, and this information is integrated for the performance. We hypothesized that the SMA is engaged in multimodal integration of information, distributed across several regions of the brain to prepare for ongoing music performance. To test this hypothesis, functional networks involving the SMA were extracted from functional magnetic resonance imaging (fMRI) data that were acquired from musicians during imagined music performance and during the resting state. Compared with the resting condition, imagined music performance increased connectivity of the SMA with widespread regions in the brain including the sensorimotor cortices, parietal cortex, posterior temporal cortex, occipital cortex, and inferior and dorsolateral prefrontal cortex. Increased connectivity of the SMA with the dorsolateral prefrontal cortex suggests that the SMA is under cognitive control, while increased connectivity with the inferior prefrontal cortex suggests the involvement of syntax processing. Increased connectivity with the parietal cortex, posterior temporal cortex, and occipital cortex is likely for the integration of spatial, emotional, and visual information. Finally, increased connectivity with the sensorimotor cortices was potentially involved with the translation of thought planning into motor programs. Therefore, the reconfiguration of the SMA network observed in this study is considered to reflect the multimodal integration required for imagined and actual music performance. We propose that the SMA network construct “the internal representation of music performance” by integrating multimodal information required for the performance.

## Introduction

Music performance requires the integration of various kinds of information, including the sensory, motor, cognitive, and emotional domains related to the performance. This information has been shown to be distributed in the brain. In this article, we sought to analyze the dynamic reconfiguration of a functional network of the supplementary motor area (SMA) during imagined music performance to better understand the mechanism of multimodal integration in music performance. Because the SMA has been associated with motor planning, we hypothesized that the SMA network is engaged in multimodal integration, thereby mediating performance planning. The SMA lies in the caudal and medial part of the superior frontal gyrus, which is a medial part of Brodmann's area (BA) 6, anterior to the primary motor cortex or BA 4 (Nachev et al., 2008; Cona and Semenza, 2017). The SMA is activated by a range of tasks that require motor programming and execution. A functional magnetic resonance imaging (fMRI) study suggested that the SMA is substantially involved in the coordination of non-homologous limbs as part of a distributed motor network (Debaere et al., 2001). More recent studies suggest that the functions of SMA are not restricted to the domain of motor control and also include non-motor functions. For example, the SMA mediates sequence processing in various cognitive domains such as action sequences, time processing, spatial processing, numerical cognition, language, and music perception, and production (Hertrich et al., 2016; Cona and Semenza, 2017). The encoding and recall of visuo-spatial sequences are associated with SMA activation (Langner et al., 2014). The involvement of the SMA in working memory has been reported in auditory, visual, spatial, and object working memories (D'Esposito et al., 1998; Hautzel et al., 2002; Gruber and von Cramon, 2003), as well as arithmetic operations (Suchan et al., 2006; Fehr et al., 2007). These observations have led to the domain-general hypothesis, which states that the SMA plays a common role in all of these various domains. Specifically, the authors hypothesized that “the SMA is involved in domain-general sequence processes, contributing as a hub to the integration of elements into a sequence” (Cona and Semenza, 2017). Because sequence processing is essential in music performance, this notion is compatible with previous studies suggesting that the SMA is a core region for music processing (Bangert et al., 2006; Cona and Semenza, 2017).

Studies have shown that the SMA is involved in the production and perception of music (Janata and Grafton, 2003; Chen et al., 2008). Music performance requires action-oriented working memory (Langner et al., 2014) and goal-directed behavioral control (Spielberg et al., 2015). To this end, an fMRI study demonstrated the involvement of the SMA in working memory during a simple music listening task (Burunat et al., 2014). Another fMRI study implicated the SMA in the anticipation of sound sequences (Leaver et al., 2009). Furthermore, the “what, when, whether model” of intentional action suggests that the SMA plays an important role in the “when” component (Brass and Haggard, 2008). It has also been demonstrated that the SMA is involved in auditory-motor transformation (Chen et al., 2009), which is required for music performance. However, several questions regarding the role of the SMA in music processing and performance remain unanswered. This article employs functional connectivity analysis to obtain insight into the role of the SMA in music.

Functional connectivity in the brain can change rapidly during task performance (Braun et al., 2015; Fuertinger et al., 2015). This dynamic reconfiguration of functional networks has been studied using various tasks, including the n-back working memory task (Cohen et al., 2014; Braun et al., 2015; Cohen and D'Esposito, 2016) and speech control (Fuertinger et al., 2015; Simonyan and Fuertinger, 2015). These studies demonstrated the ability of the human brain to reconfigure its functional network dynamically, based on current cognitive demands (Cole et al., 2013; Cohen and D'Esposito, 2016). Based on this premise, the network configuration of the SMA may be subject to dynamic change during imagined music performance if the network is involved in the task. To date, functional connectivity studies of the SMA are limited. At rest, the SMA has been shown to have connections with the primary motor cortex (M1)/primary somatosensory cortex (S1), premotor cortex (PMC), middle frontal gyrus (MFG), orbitofrontal cortex (OFC), and thalamus (Zhang et al., 2012). In this study, we evaluated a functional network involving the SMA during imagined music performance and the resting state in order to observe dynamic reconfiguration of the SMA network. If the SMA increases its functional connectivity during imagined performance, the network would be considered to be utilized for processing information needed for the performance. We predicted that the active network during imagined performance would represent processes resembling actual music performance minus processes related to motor output control. Motor output was suppressed in our experiment since participants were lying in the MRI device and instructed to remain immobile. Because of this physical state, imaging will be able to dissociate non-motor from motor processes. We also predicted that the SMA network mediating cognitive processes not directly linked to motor control is enhanced during imagined performance compared with the resting state.

## Materials and methods

### Ethical issues

All study procedures were approved by the ethics committees of Sophia University and Juntendo University and the study conformed to the tenets of the Declaration of Helsinki. All subjects provided written informed consent prior to study participation.

### Participants

We recruited 41 graduate and undergraduate music school students (mean age, 23.4 years; age range, 19–30 years). All participants were healthy, right-handed, Japanese women, without a history of neurological or neuropsychiatric disease. Students majoring in music had begun musical training at the age of 3–5 years (i.e., all participants had more than 15 years of musical training) and had actively participated in concert performances. These students specialized in classical music played on various instruments: 15 played the piano, 8 the violin, 4 the clarinet, and 14 were opera vocalists.

### Tasks

All participants completed two fMRI sessions: the first was the imagined music performance session followed by the resting-state session. Each session lasted 6 min and 40 s. During the imagined music performance session, the participants were asked to imagine the act of music performance in a concert hall as vividly as possible without performing actual movements with their eyes closed. The music “performed” was chosen freely from their repertoires. For example, pianists chose a piece of piano music (e.g., Ballade No. 1 by Frederic Chopin), violinists chose a piece of violin music (e.g., Violin Sonata No. 1 by Robert Schumann), and vocalists chose an opera aria (e.g., “Regnava nel silenzio” from Lucia di Lammermoor by Donizetti). The performance was truncated at the end of each session. In the resting-state session, the participants were instructed not to think anything in particular with their eyes closed.

### Image acquisition

#### Structural images

Whole-brain images were acquired on a Philips Achieva 3.0-T MRI scanner equipped with 32-channel head coils. High-resolution T1-weighted images were collected for anatomical reference using a 3D magnetization-prepared rapid acquisition gradient echo (MPRAGE) sequence: echo time (TE) = 3.3 ms, repetition time (TR) = 15 ms, flip angle = 10°, matrix size = 180 × 256 × 256, and voxel size = 1 × 1 × 1 mm^3^. The total image acquisition time was 3 min 31 s.

#### Functional images

Blood-oxygen-level dependent (BOLD) fMRI data were collected during the imagined music performance session and the resting-state session. A T2^*^-weighted gradient-echo-planar imaging sequence was used with the following parameters: TE = 30 ms, TR = 2,000 ms, flip angle = 90°, field of view = 240 × 240 mm^2^, matrix size = 64 × 64, number of axial slices = 33, and voxel size = 3.75 × 3.75 × 4.00 mm^3^. Each session consisted of 200 scans. The image acquisition time was 6 min 40 s.

### Preprocessing

Imaging data were preprocessed using the CONN toolbox (Whitfield-Gabrieli and Nieto-Castanon, 2012) run on MATLAB (R2016b, MathWorks, Inc.). The individual fMRI data were co-registered to the T1 images. The first four volumes were discarded and the remaining 196 volumes were subjected to preprocessing. The fMRI data were slice-timed, realigned, and subsequently normalized to the standard Montreal Neurological Institute (MNI) template as implemented in the Statistical Parametric Mapping (SPM) software platform. Image artifacts originating from head movement were processed using the ART scrubbing procedure. Signal contributions from white matter, cerebrospinal fluid, and micro head movements (six parameters) were regressed out from the data. Finally, the fMRI data were band-pass filtered (0.008–0.09 Hz) and functional images were spatially smoothed using a Gaussian filter kernel (full width at half-maximum = 8 mm) for the subsequent seed-to-voxel analysis.

### Statistical analyses

A seed-to-voxel functional connectivity analysis was performed using the CONN toolbox. The seed was either the left or right SMA. Pearson's correlation coefficients were calculated between the time course of the left or right SMA and the time courses of all other voxels in the gray matter, which provided a seed-to-voxel connectivity matrix. Positive and negative correlation coefficients defined positive and negative functional connectivity, respectively (Whitfield-Gabrieli and Nieto-Castanon, 2012). The correlation coefficients were then converted to normally distributed scores using Fisher's transformation and subsequently used in the population-level analysis. A height threshold of *p* < 0.001, uncorrected, was applied to individual voxels to define clusters. The extracted clusters were then set to *p* < 0.05 with the family-wise error (FWE) correction for results reporting.

## Results

**Figure 1:**
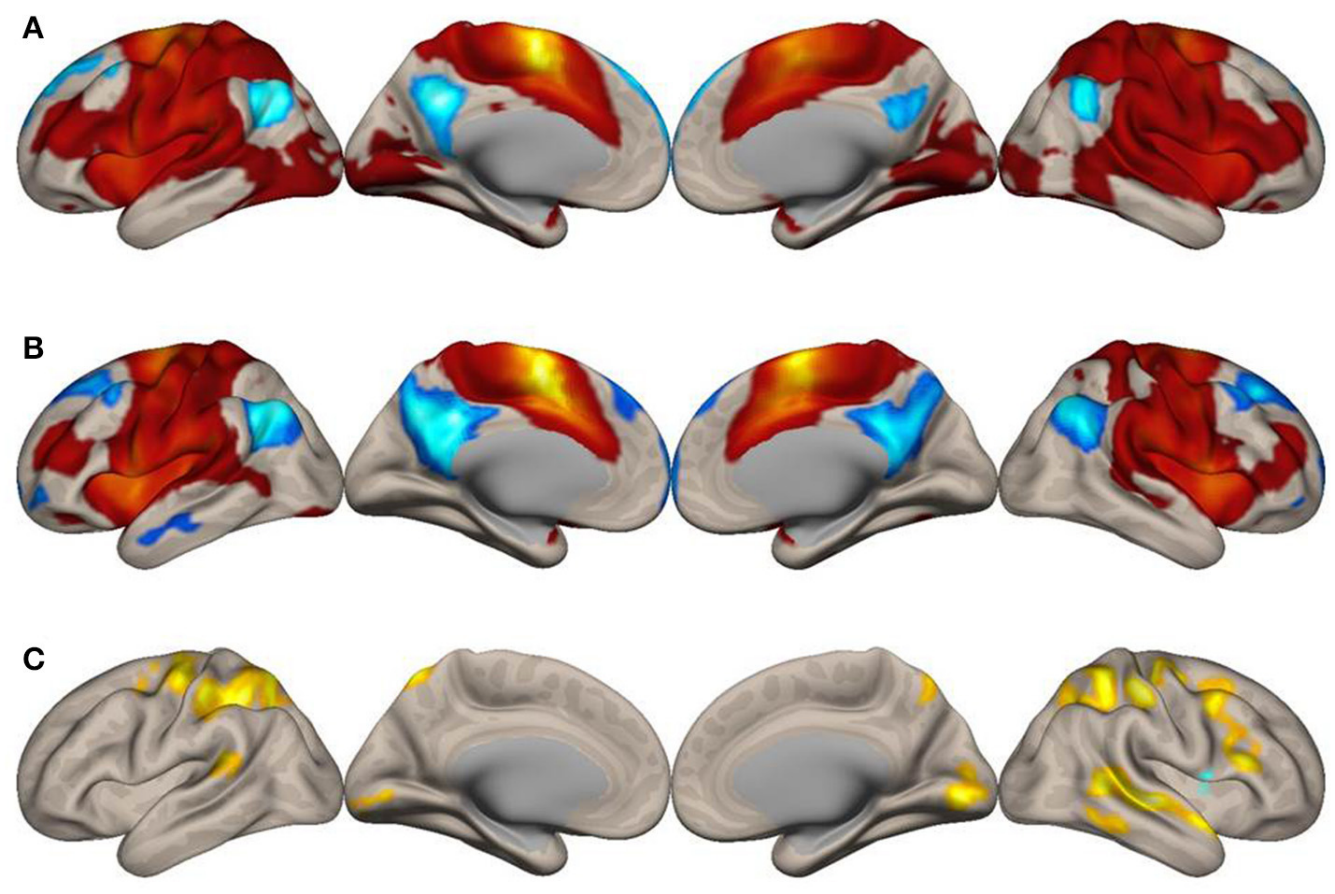
Surface maps of functional connectivity of the left supplementary motor area (SMA) as the seed region. **(A)** Imagined music performance. **(B)** Resting state. Voxels with positive connectivity are colored in red and voxels with negative connectivity are colored in blue. **(C)** Voxels with significant differences in connectivity with the left SMA between the imagined music performance and resting-state conditions are in yellow to indicate higher connectivity and blue lower connectivity during the performance condition, compared with the resting condition. The significance level was peak-voxel *p* < 0.001 (T ≥ 3.55), uncorrected, and cluster *p* < 0.05, family-wise error corrected.

**Figure 2:**
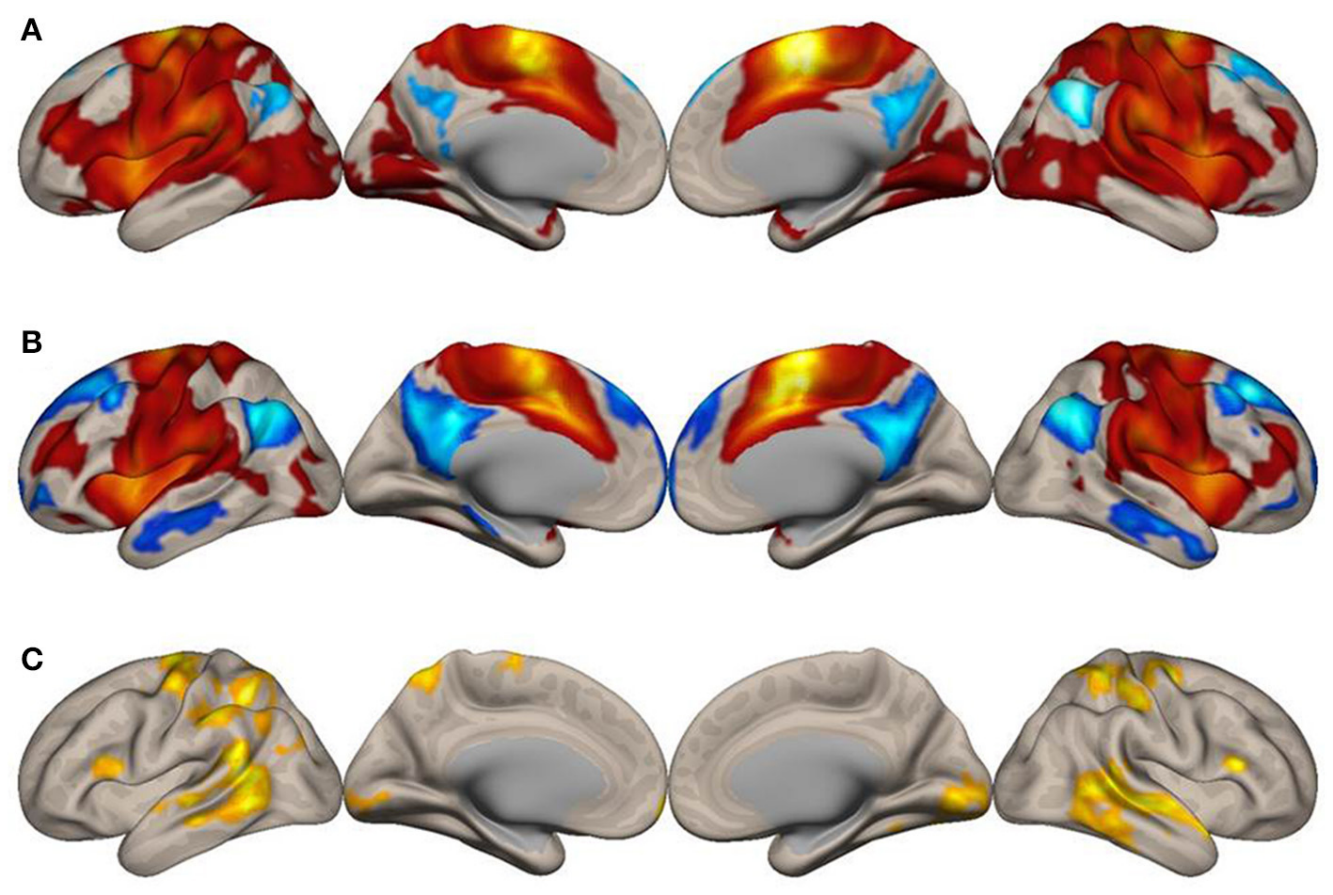
Surface maps of functional connectivity of the right supplementary motor area (SMA) as the seed region. All conventions are as in Figure 1.

To explore how the SMA network is reconfigured during imagined music performance, we analyzed functional connectivity of the SMA during both imagined music performance and the resting sate. The analysis extracted significantly enhanced functional network of the SMA. Figures 1, 2 show the results of the seed-to-voxel functional connectivity analysis. Lists of the clusters of voxels whose functional connectivity with the SMA differed significantly between the imagined music performance condition and the resting condition are given in Tables 1, 2. The results showed that the bilateral SMA exhibited enhanced functional connectivity with clusters of voxels in the superior parietal lobule (SPL), superior lateral occipital cortex (sLOC), precuneus, precentral gyrus (preCG), postcentral gyrus (postCG), supramarginal gyrus (SMG), inferior frontal gyrus (IFG), posterior temporal and occipital regions, and cerebellum during imagined music performance compared with the resting state (*p* < 0.05, FWE). The posterior temporal regions include the posterior superior temporal gyrus (pSTG), posterior middle temporal gyrus (pMTG), temporo-occipital middle temporal gyrus (toMTG), and temporo-occipital inferior temporal gyrus (toITG). Connectivity of the left SMA with the clusters of voxels in the bilateral superior frontal gyrus (SFG), MFG, and caudate nucleus and the right SMA with the clusters of voxels in the bilateral frontal pole (FP) was also increased during imagined music performance. On the other hand, the left SMA showed decreased connectivity with the voxels in the right central operculum during imagined performance compared with the resting condition.

**Table 1:** Clusters of voxels whose functional connectivity with the left supplementary motor area (SMA) differed significantly between the imagined music performance condition and the resting condition.

**Cluster (x,y,z)**	**Size**	**Cluster *p*-FWE**	**Peak *p*-unc**
(−48 −34 +48)	9147	0.0000	0.000000
(+56 −24 −04)	1648	0.0000	0.000000
(+04 −84 +00)	705	0.0000	0.000001
(−16 −70 −30)	581	0.0001	0.000001
(+18 −46 −22)	511	0.0003	0.000001
(+10 +06 +00)	501	0.0004	0.000019
(−52 −40 +16)	326	0.0052	0.000011
(−18 +10 +12)	285	0.0103	0.000019
(+58 −40 −14)	222	0.0312	0.000079
(+34 +04 +12)	197	0.0496	0.000998
*Cluster 1*			
1001 voxels covering 20% of atlas.sLOC l (Lateral Occipital Cortex, superoir division Left)			
854 voxels covering 20% of atlas.PreCG l (Precentral Gyrus Left)			
838 voxels covering 57% of atlas.SPL l (Superior Parietal Lobule Left)			
790 voxels covering 54% of atlas.SPL r (Superior Parietal Lobule Right)			
726 voxels covering 26% of atlas.MFG r (Middle Frontal Gyrus Right)			
719 voxels covering 15% of atlas.sLOC r (Lateral Occipital Cortex, superoir division Right)			
505 voxels covering 12% of atlas.PreCG r (Precentral Gyrus Right)			
491 voxels covering 13% of atlas.PostCG l (Postcentral Gyrus Left)			
438 voxels covering 14% of atlas.PostCG r (Postcentral Gyrus Right)			
396 voxels covering 42% of atlas.aSMG l (Supramarginal Gyrus, anterior division Left)			
214 voxels covering 4% of atlas.Precuneus (Precuneus Cortex)			
211 voxels covering 20% of atlas.pSMG l (Supramarginal Gyrus, posterior division Left)			
186 voxels covering 15% of atlas.pSMG r (Supramarginal Gyrus, posterior division Right)			
177 voxels covering 6% of atlas.MFG l (Middle Frontal Gyrus Left)			
166 voxels covering 24% of atlas.IFG oper r (Inferior Frontal Gyrus, pars opercularis Right)			
139 voxels covering 5% of atlas.SFG r (Superior Frontal Gyrus Right)			
131 voxels covering 24% of atlas.IFG tri r (Inferior Frontal Gyrus, pars triangularis Right)			
105 voxels covering 13% of atlas.aSMG r (Supramarginal Gyrus, anterior division Right)			
67 voxels covering 2% of atlas.SFG l (Superior Frontal Gyrus Left)			
*Cluster 2*			
394 voxels covering 34% of atlas.toMTG r (Middle Temporal Gyrus, temporooccipital division Right)			
306 voxels covering 23% of atlas.pMTG r (Middle Temporal Gyrus, posterior division Right)			
182 voxels covering 44% of atlas.pSTG r (Superior Temporal Gyrus, posterior division Right)			
115 voxels covering 41% of atlas.aSTG r (Superior Temporal Gyrus, anterior division Right)			
98 voxels covering 4% of atlas.TP r (Temporal Pole Right)			
98 voxels covering 8% of atlas.pSMG r (Supramarginal Gyrus, posterior division Right)			
*Cluster 3*			
193 voxels covering 11% of atlas.LG r (Lingual Gyrus Right)			
176 voxels covering 23% of atlas.ICC r (Intracalcarine Cortex Right)			
73 voxels covering 3% of atlas.OP r (Occipital Pole Right)			
59 voxels covering 2% of atlas.OP l (Occipital Pole Left)			
*Cluster 4*			
157 voxels covering 7% of atlas.Cereb1 l (Cerebellum Crus1 Left)			
62 voxels covering 5% of atlas.Cereb6 l (Cerebellum 6 Left)			
54 voxels covering 6% of atlas.Cereb45 l (Cerebellum 4 5 Left)			
*Cluster 5*			
176 voxels covering 29% of atlas.Cereb45 r (Cerebellum 4 5 Right)			
126 voxels covering 8% of atlas.Cereb6 r (Cerebellum 6 Right)			
*Cluster 6*			
223 voxels covering 43% of atlas.Caudate r (Caudate Nucleus Right)			
*Cluster 7*			
116 voxels covering 21% of atlas.PT l (Planum Temporale Left)			
88 voxels covering 8% of atlas.pSMG l (Supramarginal Gyrus, posterior division Left)			
*Cluster 8*			
169 voxels covering 32% of atlas.Caudate l (Caudate Nucleus Left)			
*Cluster 9*			
150 voxels covering 19% of atlas.toITG r (Inferior Temporal Gyrus, temporooccipital part Right)			
*Cluster 10*			
120 voxels covering 14% of atlas.CO r (Central Opercular Cortex Right)			

*Only the regions whose cluster size were larger than or equal to 50 were listed*.

**Table 2:** Clusters of voxels whose functional connectivity with the right supplementary motor area (SMA) differed significantly between the imagined music performance condition and the resting condition.

**Cluster (x,y,z)**	**Size**	**Cluster *p*-FWE**	**Peak *p*-unc**
(−56 −50 +12)	6977	0.0000	0.000000
(+52 −30 −04)	3030	0.0000	0.000001
(+26 −10 +60)	1971	0.0000	0.000000
(+16 −50 −30)	1835	0.0000	0.000001
(−02 +68 −12)	443	0.0009	0.000001
(+56 +26 +20)	256	0.0175	0.000001
(−50 +18 +16)	228	0.0288	0.000048
*Cluster 1*			
1193 voxels covering 24% of atlas.sLOC l (Lateral Occipital Cortex, superoir division Left)			
821 voxels covering 19% of atlas.PreCG l (Precentral Gyrus Left)			
727 voxels covering 84% of atlas.toMTG l (Middle Temporal Gyrus, temporooccipital part Left)			
565 voxels covering 53% of atlas.pSMG l (Supramarginal Gyrus, posterior division Left)			
551 voxels covering 38% of atlas.SPL l (Superior Parietal Lobule Left)			
543 voxels covering 57% of atlas.aSMG l (Supramarginal Gyrus, anterior division Left)			
352 voxels covering 25% of atlas.pMTG l (Middle Temporal Gyrus, posterior division Left)			
285 voxels covering 30% of atlas.AG l (Angular Gyrus Left)			
246 voxels covering 7% of atlas.PostCG l (Postcentral Gyrus Left)			
226 voxels covering 4% of atlas.Precuneus (Precuneus Cortex)			
132 voxels covering 23% of atlas.PT l (Planum Temporale Left)			
122 voxels covering 31% of atlas.pSTG l (Superior Temporal Gyrus, posterior division Left)			
111 voxels covering 5% of atlas.iLOC l (Lateral Occipital Cortex, inferior division Left)			
*Cluster 2*			
734 voxels covering 63% of atlas.toMTG r (Middle Temporal Gyrus, temporooccipital part Right)			
447 voxels covering 33% of atlas.pMTG r (Middle Temporal Gyrus, posterior division Right)			
367 voxels covering 47% of atlas.toITG r (Inferior Temporal Gyrus, temporooccipital part Right)			
210 voxels covering 50% of atlas.pSTG r (Superior Temporal Gyrus, posterior division Right)			
132 voxels covering 47% of atlas.aSTG r (Superior Temporal Gyrus, anterior division Right)			
116 voxels covering 12% of atlas.pITG r (Inferior Temporal Gyrus, posterior division Right)			
114 voxels covering 9% of atlas.pSMG r (Supramarginal Gyrus, posterior division Right)			
112 voxels covering 5% of atlas.TP r (Temporal Pole Right)			
50 voxels covering 12% of atlas.aMTG r (Middle Temporal Gyrus, anterior division Right)			
*Cluster 3*			
644 voxels covering 20% of atlas.PostCG r (Postcentral Gyrus Right)			
576 voxels covering 39% of atlas.SPL r (Superior Parietal Lobule Right)			
506 voxels covering 12% of atlas.PreCG r (Precentral Gyrus Right)			
81 voxels covering 10% of atlas.aSMG r (Supramarginal Gyrus, anterior division Right)			
*Cluster 4*			
288 voxels covering 17% of atlas.LG r (Lingual Gyrus Right)			
244 voxels covering 10% of atlas.OP r (Occipital Pole Right)			
227 voxels covering 15% of atlas.Cereb6 r (Cerebellum 6 Right)			
160 voxels covering 6% of atlas.Cereb1 r (Cerebellum Crus1 Right)			
147 voxels covering 17% of atlas.OFusG r (Occipital Fusiform Gyrus Right)			
115 voxels covering 15% of atlas.ICC r (Intracalcarine Cortex Right)			
106 voxels covering 17% of atlas.Cereb45 r (Cerebellum 4 5 Right)			
98 voxels covering 4% of atlas.OP l (Occipital Pole Left)			
*Cluster 5*			
250 voxels covering 4% of atlas.FP l (Frontal Pole Left)			
98 voxels covering 1% of atlas.FP r (Frontal Pole Right)			
*Cluster 6*			
104 voxels covering 15% of atlas.IFG oper r (Inferior Frontal Gyrus, pars opercularis Right)			
90 voxels covering 16% of atlas.IFG tri r (Inferior Frontal Gyrus, pars triangularis Right)			
*Cluster 7*			
171 voxels covering 22% of atlas.IFG oper l (Inferior Frontal Gyrus, pars opercularis Left)			
55 voxels covering 8% of atlas.IFG tri l (Inferior Frontal Gyrus, pars triangularis Left)			

*Only the regions whose cluster size were larger than or equal to 50 were listed*.

## Discussion

### Motor coordination

#### The superior parietal lobule

We identified increased connectivity of the SMA with the SPL and its posterior extension into the occipital cortex, the sLOC, during imagined music performance. The SPL receives input from the visual cortex and somatosensory cortex. Accordingly, the SPL processes visuospatial imagery, attention, and working memory (Schacter et al., 2012; Spreng et al., 2013). The anterior part of BA 7, a part of the SPL, has been implicated in the sequencing of hand actions (Heim et al., 2012). This area has also been previously associated with speech production, and is therefore considered to be involved in general motor sequencing. The left SPL has been associated with tool use (Garcea and Mahon, 2014). Typewriting and handwriting activate the SPL, SMG, and left premotor cortex close to Exner's area (Planton et al., 2013; Higashiyama et al., 2015). A structural equation modeling study showed that the SPL contributes via inputs to the SMA to the formation of motor imagery during a task conceiving visual or kinesthetic imagery of unpaced thumb-opposition movement (Solodkin et al., 2004). In general, the posterior parietal cortex has been associated with the planning and control of goal-directed action (Vesia and Crawford, 2012). The finding that the SPL mediates motor sequencing in the hand and speech domains (Heim et al., 2012) has important implications for its association with music processing. The SPL is consistently activated during covert musical rehearsal (Langheim et al., 2002), which is similar to the task employed in this study. The SPL is also activated during music sight-reading (Schon et al., 2002) and mental reversal of imagined melodies (Zatorre et al., 2010). Therefore, consistent with the findings of previous studies, our finding of increased functional connectivity between the SMA and SPL/sLOC during imagined music performance suggests that the SMA network mediates information processing related to intended music performance, including intended manipulation of an instrument.

#### The pre/postcentral gyrus and cerebellum

Connectivity of the SMA with the pre/postCG and cerebellum was significantly increased during imagined music performance. The preCG or motor cortex is traditionally thought to control voluntary ongoing movements (Tomasino and Gremese, 2016). Thus, our result was unexpected given that participants were not allowed to execute movements during the task. The pre/postCG or sensorimotor areas are principal regions for voluntary movement control (Tomasino and Gremese, 2016). Therefore, this result can be interpreted as follows: first, connections between the SMA and motor regions could represent a pathway involved in the translation of an intended action into a motor program. Second, it is possible that the SMA and motor cortex worked cooperatively to dissociate body movements from the imagined performance. During the imagined performance task, participants had to make conscious efforts not to move their body parts; thus, increased connectivity between the SMA and motor regions may have been utilized to inhibit involuntary body movements. Third, given that the motor cortex is involved in cognitive as well as motor processing (Leisman et al., 2016), connectivity between the SMA and motor cortex may represent an avenue of motor-cognitive processing. A recent meta-analysis identified activation of the motor cortex in response to six cognitive functional categories: motor imagery, working memory, mental rotation, social/emotion/empathy, language, and auditory processing (Tomasino and Gremese, 2016). If the motor cortex mediates cognitive processing (Tomasino and Gremese, 2016), the motor cortex would accomplish this by functional networking with the SMA and other related areas.

### The perisylvian network

In the present study, the SMA exhibited increased connectivity with the SMG during imagined music performance. The increased connectivity with the left SMG was more marked than that with the right SMG for both the left and right SMA. The left SMG is a node of the dorsal language pathway (Catani et al., 2005; Gierhan, 2013) and involved in speech production and perception as well as verbal working memory (Hartwigsen et al., 2010; Turkeltaub and Coslett, 2010; Zevin et al., 2010; Deschamps et al., 2014; Simmonds et al., 2014; Simonyan and Fuertinger, 2015). One fMRI study demonstrated that the SMG was activated during immersive second language learning in monolingual adults, and that activity was positively correlated with second-language acquisition capability (Barbeau et al., 2016). In a study of bilingual individuals, functional connectivity between the SMG and IFG was enhanced in those who became simultaneously bilingual, compared with those who became sequentially bilingual, and the degree of connectivity was correlated with the age of second language acquisition in sequential bilingual individuals (Berken et al., 2016). Music performance also requires cooperating auditory and motor processing. This is evidenced by fMRI studies indicating the involvement of the SMG in music processing using auditory and motor tasks in professional pianists (Bangert et al., 2006; Bengtsson and Ullén, 2006). The SMG is also associated with pitch memory and regulation (Zarate and Zatorre, 2008; Schaal et al., 2015a,b). Therefore, our finding of increased functional connectivity between the SMA and SMG during imagined performance is consistent with previous findings, supporting a relationship between music performance and speech production as suggested in previous studies (Koelsch et al., 2002; Brown et al., 2006). The SMG participates with the IFG in a network that mediates the abstract coding of audiovisual speech (Hasson et al., 2007). Studies suggest that the IFG, especially in the right hemisphere, mediates music syntax processing (Koelsch, 2005), and that the volume of the gray matter in the right IFG is larger in musicians than in non-musicians (Sato et al., 2015). The IFG is structurally and functionally connected with the SMG and STG (Kelly et al., 2010), forming a part of the perisylvian network for language processing (Catani et al., 2005; Xiang et al., 2010; Friederici, 2011; Gierhan, 2013). It should be noted that the connectivity of the SMA with the IFG, SMG, and STG was increased during imagined music performance. To our knowledge, our functional connectivity analysis is the first to show the recruitment of this network by the SMA during imagined music performance. Thus, it appears that the SMA contributes to music syntax processing during imagined music performance by interacting with the perisylvian network. In contrast to language syntax processing (den Ouden et al., 2012), the increased connectivity between the SMA and perisylvian network was not restricted to the left hemisphere. It seems that the network for music syntax processing is bilateral or even rightward lateralized, as indicated by rightward lateralized activation during music syntax processing (Koelsch, 2005; Musso et al., 2015). It should be noted, however, that there remains a debate concerning the laterality of syntax processing in music (Patel, 2003; Asano and Boeckx, 2015). A recent study suggests a common syntax processing role of the left IFG or the Broca's area in music and language (Kunert et al., 2015). Further investigations are needed for elucidating the network for music syntax processing.

### Visual imagery

Our analysis showed increased connectivity of the SMA with the precuneus, PCC, lateral occipital cortex (LOC), lingual gyrus, intracalcarine cortex, and OP during imagined music performance. The precuneus and PCC have been previously associated with the mental representation of visual scenes (Cavanna and Trimble, 2006; Johnson and Johnson, 2014; Tanaka and Kirino, 2016, 2017). Therefore, connecting with visual areas may enable the SMA to process mental imagery of body movements and visual scenes. The LOC includes the middle temporal (MT)/V5 and extrastriate body area (EBA). The MT/V5 mediates visual motion perception (Born and Bradley, 2005), generally showing higher responses to moving body parts than to moving objects (Spiridon et al., 2006). The EBA mediates the visual perception of body parts (Astafiev et al., 2004) and is thought to contribute to understanding goal-directed actions by representing dynamic aspects of human motion (Takahashi et al., 2008). Activity of the EBA is modulated by limb (arm and foot) movements and by motor imagery without actual movements (Astafiev et al., 2004). Based on this premise, increased connectivity between the right SMA and visual cortex is intriguing, as participants kept their eyes closed during the imaging session. Increased connectivity between the SMA and these visual areas suggests that imagined music performance includes the processing of visual scene and imagined body movements.

### Semantic and socio-emotional processing

The SMA showed increased functional connectivity with posterior temporal regions (pSTS, pMTG, toMTG) during imagined music performance. Activation of the pSTS/MTG has been associated with audiovisual semantic integration (Li et al., 2016). The pMTG is thought to be relatively specialized for semantic control (Noonan et al., 2013). Indeed, posterior regions of temporal lobe (pMTG and toMTG) contribute to semantic cognition and control (Whitney et al., 2011a,b; Jefferies, 2013; Davey et al., 2015, 2016). Semantic control and action understanding are processed by a network that includes the pMTG (Davey et al., 2015, 2016). The pMTG represents object properties (Martin, 2007), and semantic judgments about manipulable objects are represented in a network that includes the pMTG (Davey et al., 2015). The pMTG is functionally connected to regions of the frontoparietal network and shows increased activity during semantically demanding tasks (Tune and Asaridou, 2016). Accordingly, the pMTG has been associated with tool and action knowledge (Martin, 2007; van Elk et al., 2014). An fMRI study demonstrated that the pSTS was activated during the multimodal perception of nonverbal vocal and facial emotional signals and associated trait emotional intelligence with activity in the right pSTS (Kreifelts et al., 2010). In an experiment using naturalistic audiovisual movie clips, the pSTS was suggested to be a hub for social perception (Lahnakoski et al., 2012). Another study reported right hemisphere dominance for the emotional processing of scenes with social contents (Calvo et al., 2015). Consistent with this finding, the increased connectivity between the SMA and the posterior temporal regions in the present study has revealed to be rightward lateralized. Taken together, enhanced connectivity between the SMA and posterior temporal regions suggests that semantic and socio-emotional processing is included in the SMA network for imagined music performance.

### Dynamic mental representation of music performance

A major question addressed in this study was the exact role of the SMA network during imagined music performance. Our result that the SMA increased its functional connectivity with many brain regions during imagined performance is consistent with the idea that the SMA integrates multimodal information required for the performance. The left SMA showed increased functional connectivity with lateral prefrontal regions (SFG/MFG/IFG), especially in the posterior regions, during our study task. These regions have been associated with central executive functions and working memory manipulation as well as maintenance (Nee and D'Esposito, 2016). Moreover, the right lateral prefrontal regions contribute to episodic memory retrieval: The activation of the right prefrontal cortex (PFC) is a consistent finding in neuroimaging studies of retrieval from episodic memory (Henson et al., 1999; Allan et al., 2000; Nyberg et al., 2000). Therefore, the increased connectivity between the SMA and the lateral prefrontal regions, especially in the right hemisphere, suggests that, under top-down control by the central executive system, the SMA construct “the internal representation of music performance” for the upcoming performance by integrating multimodal information represented in several brain regions. Just as working memory is not localized to a single brain region (D'Esposito, 2007), the executive imagery for music performance may be an emergent property of the interaction between the PFC and the SMA. This view is consistent with the rostro-caudal gradient organization theory of PFC functions, in which the posterior PFC represents behavioral and motor information (Badre, 2008; Depue et al., 2016). Complex cognitive control and time estimation, which are also required in music performance, engage the overlapping brain regions including the SMA, PMC, SPL, and precuneus (Radua et al., 2014). Our result is consistent with their meta-analysis. The enhanced network of the SMA during imagined music performance also include the temporal and occipital regions. The inclusion of these regions into the network indicates that the network mediates multimodal integration of the information needed for performance, specifically, the integration of visual imagery, syntactic, semantic, and socio-emotional information into motor imagery. Because various motor imagery tasks activate brain regions including the SMA, SPL, IFG, and preCG (Hanakawa et al., 2008; Hétu et al., 2013), our results suggest that the SMA network processes motor imagery. Therefore, we believe that information processing by the enhanced SMA network mediates multimodal integration into motor imagery for imagined music performance. Regarding functional networks, however, no comparison has ever been made between imagined and actual music performance. To date, an fMRI study reported that imagined and actual playing of short piano pieces in pianists activated brain regions in common: the sensorimotor, premotor, posterior parietal, and visual cortical areas, precuneus, and cerebellum (Meister et al., 2004). Among these regions, the sensorimotor and posterior parietal cortical areas were activated more during actual playing, which suggests that motor execution required the higher level of visuomotor integration.

## Conclusion

We have characterized dynamic network reconfiguration of the SMA for imagined music performance. Functional connectivity between the SMA and regions distributed over the parietal, temporal, occipital, and frontal cortices as well as the cerebellum is increased during imagined music performance, compared with the resting state. The SMA processes multimodal information including visuospatial, motor, syntax, semantic, and socio-emotional information relevant to intended music performance. We propose that the SMA network dynamically construct “the internal representation of music performance” by integrating multimodal information into motor imagery required for the performance.

## Author contributions

ST and EK planned and conducted all the experiments. ST analyzed the data and wrote the manuscript.

### Conflict of interest statement

The authors declare that the research was conducted in the absence of any commercial or financial relationships that could be construed as a potential conflict of interest.
